# Supplementary material for: Effects of Online, Asynchronous Education Modules on Migraine Severity and Elimination Diet Use Among Higher Education Students: An Observational, Pilot Feasibility Study
Source: Nutrients. 2025 Jul 25;17(15):2432. doi: 10.3390/nu17152432 (PMC12348680; doi:10.3390/nu17152432)
Supplement: Supplementary file 1 [file nutrients-17-02432-s001.zip › nutrients-3701368-supplementary.pdf]

Supplementary Materials

Supplementary Material A: Pre-Education and Post-Education Assessments 2

Supplementary Material B: Two Month Follow-Up Survey 7

Supplementary Material C: The Educational Course 13

Table S1. Knowledge and Self-Efficacy at Time 2 43

Table S2. Change in MIDAS Scores by Medication Change 44

Table S3. Barriers to Implementing the Elimination Diet at Time 2 45

Supplementary Material A

Pre-Education and Post-Education Assessments

I. <u>Knowledge of Migraines and Headaches</u>

Q1: Migraines have the same symptoms as other headaches.

[True/False] (F)

Q2: For migraines, the source of the pain is psychological rather than physical.

[True/False] (F)

Q3: Migraines are more common in females than males.

[True/False] (T)

Q4: Migraines are caused by the activation of a mechanism deep in the brain that leads to the release of pain-producing inflammatory substances around the nerves and blood vessels of the head.

[True/False] (T)

Q5: Migraine pain typically occurs on both sides of the head.

[True/False] (F)

Q6: Migraine is a whole-body disorder that can affect virtually every part of the body.
[True/False] (T)

Q7: Migraine affects every person and every body differently. [True/False] (T)

Q8: There are no prescription medications (i.e., those that require a prescription from a doctor) available that can work in the short term to relieve migraine symptoms. [True/False] (F)

Q9: The over-use of medications designed to provide short-term relief for migraine can lead to more headaches. [True/False] (T)

Q10: Prescription medications are available that can help to prevent migraine attacks from occurring. [True/False] (T)

Q11: For the following symptoms, choose the most correct option. Note that this is not a complete list of migraine symptoms.

	(1) Can be associated with migraine	(2) Is never associated with migraine
Nausea		
Seizure		
Sensitivity to light		
Sensitivity to sound		
Sinus pain and pressure		
Heat flashes and chills		
Frequent urination		
Dizziness, temporary paralysis		

Numbness and tingling		
Hallucinations and visual disturbances		
Earaches		
Jaw pain and yawning		
Gastrointestinal symptoms		
Sensitivity to smell, touch, motion and light		
Joint pain and stiffness		
Depression, anxiety, memory lapses and difficulty concentrating		
Sensory disturbances (often visual) called aura		
Pulsating headache		
Pain is often aggravated by routine physical activity		

Q12: A migraine episode or attack typically lasts:

A. Seconds to minutes

B. Minutes to hours

C. Hours to several days

D. Days to weeks

II. Knowledge of the Elimination Diet

A. Questions on general concepts (T/F):

1) Migraines may be triggered by certain types of food. (T)
2) One approach to decrease migraines is to avoid food and beverage triggers. (T)
3) Dietary modification has no role in preventing migraines. (F)
4) Identifying migraine/headache triggers can be challenging because it may take several hours to days until their impact is felt. (T)
5) It is not difficult to identify triggers despite the fact that migraines often follow exposure to more than one trigger / a combination of triggers. (F)
6) Migraine dietary triggers act in isolation and do not act in concert with other factors/triggers that are difficult to identify and control. (F)
7) Skipping or delaying meals can be a migraine trigger. (T)
8) Even when dietary modification is done properly, but fails to control migraines satisfactorily, this does not mean that dietary triggers aren’t important. (T)

B. Questions on different components of the diet: (T/F)

1) The elimination diet has two stages: elimination and reintroduction. (T)
2) If a person is allergic to a food item, that food item may be a migraine trigger for them. (F)
3) The specific way in which foods trigger migraines is well-known. (F)
4) The effect of each dietary trigger is dose-related: the more one consumes, the worse the migraine will be. (T)

5) When starting an elimination diet, individuals should strictly avoid all potential dietary triggers. (T)

<u>C. Questions on specific dietary triggers:</u>

<u>T/F</u>

1) Caffeine, or food items that contain caffeine, can ward off an acute migraine episode and is a safe long-term reliever (for example beverages such as coffee, tea, colas, Mountain Dew, Dr. Pepper). (F)
2) Natural flavors and fermented items are not a potential source of MSG. (F)

3) Are the following foods potential triggers for migraine?

	**Yes**	**No**
Chocolate		
Processed meats		
Cheese		
Dairy, such as milk, cream, butter		
Nuts, nut butter, and nut extracts		
Fruits high in tyramine, such as bananas, raisins, dried fruits (preserved with sulfites), raspberries, red plums, papayas, passion fruit, figs, dates, and avocados		
Fresh yeast-risen baked goods (less than one day old)		
Aspartame and other artificial sweeteners, such as sorbitol or sucralose (Splenda)		

Supplementary Material B

2 Month Follow Up Survey

I. Knowledge, Efficacy and Adherence

**1. I have incorporated the elimination diet into my everyday life since completing the workshops.**					
(1) Completely disagree	(2) Somewhat disagree	(3) Neutral	(4) Somewhat agree	(5) Completely agree	
Please explain why or why not:					
Have you started any new medications since beginning the study? Yes/No<br>If so, please list:					
**2. I feel that I have been well-informed about ways in which I can be most successful with the elimination diet.**					
(1) Completely disagree	(2) Somewhat disagree	(3) Neutral	(4) Somewhat agree	(5) Completely agree	
**3. In general, the education from this study has helped me improve my diet.**					
(1) Completely disagree	(2) Somewhat disagree	(3) Neutral	(4) Somewhat agree	(5) Completely agree	

**4. In general, my nutrition knowledge has improved since being on the elimination diet.**					
(1) Completely disagree	(2) Somewhat disagree	(3) Neutral	(4) Somewhat agree	(5) Completely agree	(6) N/A
**5. Following the elimination diet has improved my eating habits in general.**					
(1) Completely disagree	(2) Somewhat disagree	(3) Neutral	(4) Somewhat agree	(5) Completely agree	(6) N/A
**6. I find the benefits of the elimination diet for improving my migraine symptoms outweigh the inconveniences that come with having a restricted diet.**					
(1) Completely disagree	(2) Somewhat disagree	(3) Neutral	(4) Somewhat agree	(5) Completely agree	(6) N/A
**7. I would recommend the elimination diet to other people with migraines.**					
(1) Completely disagree	(2) Somewhat disagree	(3) Neutral	(4) Somewhat agree	(5) Completely agree	
**8. The elimination diet does a good job in keeping my migraine symptoms under control.**					
(1) Completely disagree	(2) Somewhat disagree	(3) Neutral	(4) Somewhat agree	(5) Completely agree	(6) N/A

II. Potential Barriers to Following the Elimination Diet

A. Social and Emotional

Please skip this section if you did not try the elimination diet

**9. Social situations, like going out with friends or family, make it harder for me to follow the elimination diet.**				
(1) Completely disagree	(2) Somewhat disagree	(3) Neutral	(4) Somewhat agree	(5) Completely agree
**10. Following the elimination diet makes me anxious.**				
(1) Completely disagree	(2) Somewhat disagree	(3) Neutral	(4) Somewhat agree	(5) Completely agree
**11. When my symptoms are in control, I sometimes stop following the diet more than usual.**				
(1) Completely disagree	(2) Somewhat disagree	(3) Neutral	(4) Somewhat agree	(5) Completely agree
**12. Following the elimination diet makes me feel down or depressed.**				
(1) Completely disagree	(2) Somewhat disagree	(3) Neutral	(4) Somewhat agree	(5) Completely agree
**13. When I am stressed or feeling ill, I stop following the elimination diet more than usual.**				
(1) Completely disagree	(2) Somewhat disagree	(3) Neutral	(4) Somewhat agree	(5) Completely agree

**14.** **I find the elimination diet to be much more difficult than I expected it to be.**				
(1) Completely disagree	(2) Somewhat disagree	(3) Neutral	(4) Somewhat agree	(5) Completely agree
**15.** **Traveling for pleasure/work has introduced a challenge in following the elimination diet.**				
(1) Completely disagree	(2) Somewhat disagree	(3) Neutral	(4) Somewhat agree	(5) Completely agree

B. Cost, Inconvenience:

Please skip this section if you did not try the elimination diet

With regard to following the elimination diet, it can be difficult to….

**1. Find something to eat.**				
(1) Completely disagree	(2) Somewhat disagree	(3) Neutral	(4) Somewhat agree	(5) Completely agree
**2. Eat out for fear of contamination.**				
(1) Completely disagree	(2) Somewhat disagree	(3) Neutral	(4) Somewhat agree	(5) Completely agree

**3. Spend a lot of time planning meals.**				
(1) Completely disagree	(2) Somewhat disagree	(3) Neutral	(4) Somewhat agree	(5) Completely agree
**4. Read food labels and shop at special stores.**				
(1) Completely disagree	(2) Somewhat disagree	(3) Neutral	(4) Somewhat agree	(5) Completely agree
**5. Stick to my grocery budget.**				
(1) Completely disagree	(2) Somewhat disagree	(3) Neutral	(4) Somewhat agree	(5) Completely agree
**6. Find foods I can eat because of my migraines.**				
(1) Completely disagree	(2) Somewhat disagree	(3) Neutral	(4) Somewhat agree	(5) Completely agree
**7. Follow because it's expensive.**				
(1) Completely disagree	(2) Somewhat disagree	(3) Neutral	(4) Somewhat agree	(5) Completely agree
**8. Follow because it's restrictive.**				

(1) Completely disagree	(2) Somewhat disagree	(3) Neutral	(4) Somewhat agree	(5) Completely agree
9. **Throw away and/or donate my food and ingredients that do not follow the elimination diet.**				
(1) Completely disagree	(2) Somewhat disagree	(3) Neutral	(4) Somewhat agree	(5) Completely agree
**10. Plan out my meals, read labels, and cook meals with specific restrictions due to school and life responsibilities.**				
(1) Completely disagree	(2) Somewhat disagree	(3) Neutral	(4) Somewhat agree	(5) Completely agree
**11. Implement the elimination diet because of the fixed student meal plan.**				
(1) Completely disagree	(2) Somewhat disagree	(3) Neutral	(4) Somewhat agree	(5) Completely agree

Do you have any other comments on this study or issues that you encountered? (Free text)

Supplementary Material C

The Educational Course

<u>I. Module 1:</u>

Page 1: What is Migraine? Part 1

-Start by going to: PollEv.com/amandajan966. Respond to the poll to contribute to the word cloud:

1) What is a headache?

- A type of pain that affects the head, scalp, or neck.
- Headaches are divided into 2 categories: primary headaches and secondary headaches.

2) What are primary and secondary headaches?

- Primary headaches are not caused by another underlying medical condition. Common types of primary headaches include migraine- and tension-type headaches.
- Secondary headaches are caused by an underlying medical condition. Examples include medication overuse, infections, injuries, blood vessel disorders, etc.

3) What is Migraine? What are the symptoms?

- Migraine is a type of primary headache that can cause severe pain and may last for hours to days. Migraines can occur in episodes, or on a regular, frequent basis. Migraines can interfere with daily activities.
- Headaches are only one symptom of migraine. Certain changes in brain activity that affect the brain and surrounding tissues can cause a range of symptoms, including nausea, increased sensitivity to light, sound, and smells, dizziness, extreme fatigue, etc. Migraines can affect virtually any part of the body. They affect each person differently. Migraine can be **chronic** (happening 15 + days/month, for 3 + months, with at least 8 days/month having migraine features, or **episodic** (which is less frequent).

Page 2: What is Migraine? Part 2

1) What is an aura?

- Some people with migraines also experience a phenomenon called aura, which is a set of symptoms that affects the nervous system and can occur before or during a migraine headache. About 15% to 20% of people who experience migraines have auras. Aura can include visual, sensory, and/or motor symptoms:

Visual

- Visual (flashing lights, wavy lines, blind spots, temporary visual loss, etc)

Sensory

- Numbness or tingling skin
- Changes in smell or taste.
- Ringing in your ears (tinnitus)
- Dizziness (vertigo)
- A "funny" feeling

Motor

- Weakness on one side of the body
- Speech changes.

2) What are the four stages of a migraine?

- The four stages are the **prodrome**, **aura**, **headache**, and **postdrome**. Not everyone with migraines experiences all four phases. The timing of the phases can vary from person to person.

  - Prodrome: Can occur 1-2 days before the other phases. Some examples are changes in mood, energy levels, or behavior, yawning, neck stiffness, increased thirst, etc.

  - Aura: Specific neurological symptoms, such as the visual, sensory, or speech disturbances described previously. Auras may last for a few minutes to an hour and may occur before or during the headache phase.

- Headache: May be characterized by severe, throbbing pain, often on one side of the head. Other symptoms may include nausea, vomiting, and sensitivity to light and sound. The headache phase can last for a few hours to several days.

- Postdrome: May involve feelings of exhaustion, confusion, or mood changes. Some describe it as a "hungover" feeling after the headache phase has resolved.

6) How to differentiate migraines from tension headaches?

- Both can be defined by head pain that starts gradually and can vary in intensity. Nasal congestion and light sensitivity can be common for both; however, nasal congestion is not the cause of both these types of headaches.

- Migraines cause moderate to severe, throbbing pain, often on one side of the head. As mentioned above, they can be accompanied by other symptoms, such as nausea, vomiting, and sensitivity to light and sound. Tension headaches do not typically have these additional symptoms and usually cause less pain than a migraine. Tension headaches may feel more like tight pressure, usually on both sides of the head.

Page 3: Risk Factors for Migraines

-First, let's review what we learned in the last module: For optimal viewing, you should expand to Full Screen Mode by clicking the icon in the top right of the Nearpod window. You should not need to make an account for any other sites. If you are asked to enter your name for an activity, please use your study ID.

1) Who gets migraines? What are the risk factors?

- There are several factors that can increase the risk of developing migraines, including

+ Family history: See #2 below

+ Gender: Women are more likely to get migraines than men.

+ Age: Migraines are most common in people between the ages of 25 and 55, although they can occur at any age.

+ Lifestyle factors: Certain lifestyle factors, such as lack of sleep, high-stress levels, or irregular eating habits, may increase the risk of migraines.

+ Hormonal changes: Fluctuations in hormone levels, such as those that occur during the menstrual cycle or menopause, may trigger migraines in some people.

+ Certain medications: Some medications, such as oral contraceptives and vasodilators, can increase the risk of migraines.

- It is important to note that not everyone with these risk factors will develop migraines, and some people who get migraines may not have any known risk factors.

2) Are migraines hereditary?

- Migraines tend to run in families, which suggests a genetic component to the condition. Studies have shown that people who have a family member with headaches are more likely to develop the condition themselves, especially if the family member is a first-degree relative (such as a parent or sibling).

- It is important to note that having a family history of migraines does not guarantee you will develop the condition. Not everyone who gets migraines has a family history of the condition. There are also other factors that play a role in the development of migraines.

-Discussion Question: ***What factors do you feel contribute to your migraines?*** *Have you noticed controllable factors that impact when you have migraines? If so, what are they?*

Page 4: What Causes a Migraine?

1) What causes a migraine?

- The exact cause of migraines is not fully understood. Migraines are thought to be related to changes in the trigeminal nerve pathway in the brainstem, a major pain pathway in the brain and upper spinal cord of the neck (this is why neck pain can be a common symptom of migraine). When you sense a headache, it’s because specific nerves in this brainstem pathway send pain signals to the central brain and up to the cortex. The signals lead to a release of inflammatory chemicals from the nerve endings and blood vessels around the brain.

- Sensitivity to stimuli that normally should not hurt, such as light and sound, may occur because of increased activity in the thalamus, the part of the brain that processes sensory stimuli.

- The thalamus may also be involved in causing dizziness associated with some migraines. Another area of the brain involved in symptoms of dizziness is the temporoparietal junction, which houses areas of the brain that process information from multiple senses to help keep our balance.

- Some molecules, such as calcitonin gene-related peptide, may be involved in causing migraines. These molecules may also be involved in the dizziness that some people experience with migraines. Their role in causing migraines, however, is still being investigated.

2) What triggers a migraine?

- A variety of factors can trigger migraines, and these triggers can vary from person to person. Some common triggers for migraines include:

    - Hormonal changes: Migraines can be common for women during their menstruation cycles. This is due to a decrease in estrogen levels. Birth control pills and hormonal replacement therapies can affect hormonal changes. Since estrogen does not generally fluctuate in young girls before puberty or post-menopausal women, migraines are typically worse between puberty and menopause.

    - Environmental factors: bright lights, loud noises, smoke, or strong smells

    - Barometric pressure & weather changes: approaching storms, heat, humidity, air travel, high altitude

    - Stress: a common trigger for migraines. Certain chemicals in the brain are released during stressful events and trigger the natural “flight or fight” response. These chemicals can cause changes in your brain chemistry and blood flow which can lead to the development of a migraine. Stress can also cause muscle tension, leading to the constriction of blood vessels in the head and neck, contributing to the development of a migraine. Additionally, stress may lead to unwanted lifestyle changes that can trigger migraines:

- Daily use of pain-relieving medications: certain medicines are meant to relieve acute headache pain, but if used too often, they can cause rebound headaches.
- Sleep: Sleep deprivation, oversleeping, or any changes in sleep patterns
- Skipping meals or changes in eating habits
- Physical factors: such as poor posture or eyestrain, bending over, overexertion during exercise/weight lifting, etc.
- Drinking alcohol.
- Diet: Certain foods and drinks, such as alcohol, caffeine, aged cheeses, etc

- *It is important to note that not everyone who gets migraines will have the same triggers, and some people may have multiple triggers.*

3) Trigger level vs. Threshold

- Two factors determine whether or not the migraine mechanism is activated, and if so, to what degree. Understanding these two important determinants and how they relate to one another can help control migraines.
    - Trigger level: Triggers vary from individual to individual.
    - Threshold: a preset level of input of triggers at which a migraine will be activated. The migraine threshold varies from one person to another and is largely genetically determined. If you have low migraine thresholds, it means that you are more susceptible to headaches and other symptoms of migraines. Especially if migraine runs in your family, then the thresholds are lower for you. For people who do not have frequent and major headache problems, the thresholds are higher; therefore, they may experience fewer and milder migraines.
    - The higher the triggers level rises, the more fully activated the migraine will be and the greater the symptoms. Once triggered, migraine symptoms can last for a few seconds, minutes, hours, to days.

4) How do triggers add up?

- Some migraine triggers are unavoidable while others are avoidable. For example, unavoidable triggers include barometric pressure changes, family history/genetics, and some stressful situations around you. Avoidable triggers include sleep deprivation, skipping meals, and not exercising.
- It is possible to decrease the probability and severity of migraine headaches by reducing triggers that you have some control over.
- Recognizing when unavoidable triggers and avoidable triggers are stacked and modifying the controllable risk factors can be helpful. For example, a graduate student may study on their computer all day without any intentional breaks and go to sleep at different times each night. This may lead to limited cooking time, so their diet may consist of processed meat and to-go frozen meals. To make matters worse, they are having their menstrual period, and the weather has been stormy. All of these triggers combined cause this student to experience a migraine. However, by themselves, the unavoidable triggers might not reach the preset migraine threshold and cause this student a migraine. In other words, if this student can schedule more breaks, go to sleep at the same time every day, and avoid migraine-triggering foods, they might not have a migraine.

-Complete this warm-up activity before continuing to the next module! For optimal viewing, you should expand to Full Screen Mode by clicking the icon in the top right of the Nearpod window. You should not need to make an account for any other sites. If you are asked to enter your name for an activity, please use your study ID.

Page 5: The Elimination Diet

An elimination diet can be a useful tool for people who experience migraines. It helps identify potential food triggers that may be contributing to migraines. By eliminating certain foods from your diet and then gradually adding them back in, you can determine if any of them are triggering migraines. This can be especially useful if you have not been able to identify any other clear triggers.

It's important to note that an elimination diet should only be undertaken if you do not have any other critical health conditions aside from migraines. It should be implemented under the guidance of a healthcare provider, as it can be a complex process and may not be suitable for everyone.

Additionally, it's important to be aware that while an elimination diet may help identify trigger foods, it may not cure migraines. Other approaches, such as medication and lifestyle changes, may also be necessary for managing migraines.

Note: Our elimination diet education program does not have detailed instructions on how to manage migraines using other approaches, including medications. Please seek other trusted resources and medical advice from your primary care physician and specialists

-Discussion Question:

Discuss your level of agreement with the following statements:

1. *An elimination diet is an effective way to minimize the frequency of migraines.*
2. *Dietary triggers are a well-known cause of migraines.*

Page 6: The Elimination Diet in Migraine

After reading the prior section, you might think that you have tried to avoid some common migraine food triggers (ex: chocolate, wine, etc.) in the past, but your migraines still have not improved.

Why do you think it is still helpful to do an elimination diet? Why do you think dietary triggers go unrecognized?

Here are some reasons:

1) It is common to find articles and resources listing a few common migraine-triggering foods, such as chocolate, red wine, processed meat, and Chinese take-outs. However, these ingredients are only the tip of the iceberg. Many other ingredients can trigger migraine symptoms.

2) Most people with migraines tend to consider some foods not triggers because of the potential delay of hours— up to a day or two—between eating the food and experiencing a migraine. You might not feel migraine symptoms immediately, so you continue eating the same type of food.

3) This is tricky: you might notice a food that triggers a migraine today but didn't trigger any migraine symptoms in the past. Obviously, it makes sense for us to conclude that this type of food is not a trigger. Remember the concept of the migraine threshold! Let's use the student example again: the same student just received a scholarship offer and is currently on break. Therefore they are not under any school pressure, so they decide to meal prep, exercise every day, and go to sleep at the same time each night. During this break, even Chinese take-out food doesn't give them a headache or any other migraine symptoms. They happily decided that the Chinese food, including MSG and other ingredients, might not be a trigger. Unfortunately, the good days of high pre-set migraine threshold fools this student and many individuals with migraines.

4) It's difficult for patients with chronic migraines to appreciate and fully recognize the full extent of dietary triggers due to the chronic nature of the migraines. Many find it hard to believe that dietary triggers can lead to migraines because they have had migraines for so long.

5) Some people with migraines notice the migraine started when there was no prior change in diet, so "it can't be the diet that causes migraines." But remember: dietary triggers are only 1 part of the list of factors that can contribute to the probability of migraines.

6) Many have tried to eliminate food triggers in the past, but it didn't work. Many people eliminate only a few items for a short period. However, adequate time is needed to discover food triggers (even during high-threshold or low-threshold days).

7) Some people have tried avoiding all commonly reported migraine triggers in their diet for an extended period, but they still experience migraines and eventually give up on dietary approaches. It's important to remember that dietary triggers alone are insufficient to keep the overall trigger level below the threshold. Preventive migraine medications and a consistent focus on lifestyle-friendly factors, such as regular sleep patterns and frequent breaks, are also necessary to avoid exceeding the trigger

threshold. Despite this, avoiding dietary triggers remains crucial for the efficacy of medications and other approaches.

Use the 24-Hour Diet Recall Template (linked below) to detail what you ate in the last 24 hours. The instructions are on the template document.

This is for your own educational purposes. You do not need to submit your document to Canvas.

II. Module 2:

-Do this warm up before starting the module: For optimal viewing, you should expand to Full Screen Mode by clicking the icon in the top right of the Nearpod window. You should not need to make an account for any other sites. If you are asked to enter your name for an activity, please use your study ID.

Page 1: Elimination Diet: Phases

The diet consists of 2 stages:

Note: The elimination diet takes 2 months in total: 1 month for the Elimination stage and 1 month for the Reintroduction stage.

1) The Elimination stage:

   a) Ideally, it's best to do this stage for 1 month. The 1 month will give enough time to observe the potential delayed reaction to some food triggers and reactions to food triggers.

   b) During this stage, individuals should avoid all commonly reported food triggers (mentioned in the subsequent section) and substitute them with other ingredients.

   c) It is important to keep a record of your daily food intake and jot down any headaches and migraine-related symptoms (dizziness, tingling, nausea, etc.). Note: Please make sure that you and your doctor are aware and agree that non-headache symptoms, such as nausea, are caused by migraines, not other medical conditions.

   d) Substitute each ingredient with other suggested migraine-friendly ingredients. Trust in the process that, over time, you will become creative with creating simple and practical recipes based on the listed migraine-friendly ingredients.

e) We will provide you with a weekly sample of recipes and a cooking website consisting of recipes that use only migraine-friendly ingredients.

2) The Reintroduction stage:

a) After the elimination diet for 1 month, pick your favorite item and consume it daily for 4 days. If the item gives you a headache, consider it a food trigger. If you do not have a migraine, consider it a safe food, at least in the quantities you've consumed up to this point, without experiencing any other triggers at the same time.

b) Then move on to the next item that you miss on the list until you sort out all your possible food triggers. Don't try to add back items that you don't really care about or items that are not good for your health anyway (such as processed meat).

c) Don't add back caffeine because it's only a quick fix for your headaches and can cause rebound headaches (more explanation on this item in the later section).

d) When you reintroduce cheese back to your diet, it might be easier for you to tolerate younger cheese varieties, such as fresh goat cheese, provolone, and mascarpone.

e) Make a list of items that cause you headaches. Be patient because it might take a couple of days for some items to trigger a migraine.

f) Add lime juice, white wine, onions, and vinegar slowly back into your diet. One at a time!

g) Remember the "good" (low total trigger load) vs. "bad" (high total trigger load) days: onions may not trigger migraine on a good day, but might trigger it on another day. This is why you allow a minimum of 4 days to add back in each item.

h) During this stage, please don't add multiple items during each 4-day period. Only 1 item at a time so you can discover your food triggers. The more food triggers you try to add within the same period, the more likely you will suffer from a headache. The same goes for the quantity of potential food triggers consumed.

-Play this matching game before reading the next section. Tap or click to highlight the first word, then tap a second word that you think is a good substitute for that food item:

For optimal viewing, you should expand to Full Screen Mode by clicking the icon in the top right of the Nearpod window. You should not need to make an account for any other sites. If you are asked to enter your name for an activity, please use your study ID.

Page 2: Elimination Diet: Nuts and Bolts

Note: This is a long list, so please feel free to read it at your own pace and take breaks as needed!

What are potential food triggers?

Elimination Diet: Nuts and Bolts

-This education section discussing each trigger foods is pretty long, you might want to break it in half (have a Part 1 and Part 2)

Explanations:

1) Caffeine:

-Any caffeinated beverage—coffee, tea (black, green, oolong tea), iced tea, chocolate drinks, coke or other caffeine-containing sodas, and energy drinks—should be completely and permanently avoided. Even decaffeinated coffee or tea contains a small amount of caffeine. Elimination of caffeine tends to cause withdrawal effects, including rebound headaches, for up to a few weeks in some people; stay strong since the long-term benefits are worth it.

-Why? You often hear and come across articles recommending caffeine as a quick way to eliminate headaches. It's certainly true…but only for a short time. Caffeine works by constricting

blood vessels, which are dilated during migraine headaches. As a result, fewer pain stimuli and chemicals that cause the pain are released. However, blood vessels will become desensitized over time and dilate even more, causing rebound headaches.

-This is by far the most difficult item for people with migraines to eliminate. If you cannot eliminate decaffeinated beverages, at least they are still better than caffeinated ones.Remember, caffeine elimination may not only lead to fewer migraines, but may also improve energy, mood, sleep, heartburn, etc.

-Substitute with

+ Herbal tea (there’re so many options)

+ Caffeine-free beverages (ex: caffeine-free cokes)

+ More options are listed on the website: https://thedizzycook.com/vm-coffee/

-Powerful tips:

+ Having enough sleep and consistent sleep patterns may make it easier to eliminate coffee

+ Take a 10-min nap when you feel tired

+ Go for a walk and do exercise when your energy dips

+ Schedule intentional mini-breaks during long sessions of working and studying

+ Switch up activities to avoid boredom

+ Stay mindful and present in everything you do without thinking about other things. Allowing your attention to wander can exhaust your energy and make it harder to focus.

2) Chocolate:

-Why? Chocolate and anything made with cocoa contain caffeine. They also contain other potential migraine-triggering chemicals, including theobromine and phenylethylamine. White chocolate is safer, but carob-containing items might be tricky. Chocolate may be hard to avoid, but there are also a variety of other sweets that you can have.

-Substitute with:

+ Caramel

+ Any other desserts other than chocolate or chocolate-containing items

3) Monosodium Glutamate (MSG):

-Sometimes it may feel like the tastiest items also trigger migraines. MSG is another one on the list. Remember, migraines were triggered for our *previous college student after having Chinese food*. Well, it is rich in MSG—the secret ingredient in most Asian food, which is why it tastes so delicious.

-Why?

+ Surprisingly, MSG can be disguised under different names. Glutamate—a natural amino acid that makes up part of your body's protein---creates or enhances the umami flavor.

+ The U.S. Food and Drug Administration considers MSG as "natural," so MSG might be present in foods labeled "all natural" and "no artificial ingredients."

+ Items such as veggie burgers, croutons, bread crumbs, seasoned salt, bouillons, protein concentrates, supplements, and even shakes can contain MSG.

-Substitute with:

+ spices and natural herbs, such as garlic, curries, ginger, horseradish, mustard seeds, cinnamon, vanilla, honey, pepper, cumin, thyme, etc. Get creative!

-Powerful tips:

+ Avoiding MSG can be tricky, so we will show you how to read labels in the subsequent section

+ BE AWARE: even though ingredients are listed in the order of decreasing quantity on food labels, MSG can be a potent migraine trigger that should be avoided

+ The best way to avoid MSG is to use fresh ingredients. The simpler the food, the better.

+ Here's the list of sources rich in MSG and glutamate:

4) Processed Meats and Fish:

-Why?

+ Meats, seafood, and fish that are aged, fermented, pre-marinated, smoked, canned, cured, tenderized, or preserved with nitrites or nitrates can be common dietary migraine triggers.

+ Beware of sausages (not all contain nitrites, but some might have MSG, and tyramine, another powerful trigger), salami, pepperoni, bologna (lunch meats with nitrates), beef jerky, certain hams, bacon, pates, smoked salmon or trout, caviar, anchovies, and pickled fish (such as herring)

+ Tyramine and glutamate tend to accumulate with aging and ripening, so please enjoy fresh food as much as you can!

-Substitute with:

+ Processed food is not recommended for overall health. They can be linked to increased cancer incidence, so try to avoid them as much as possible.

5) Cheese and other dairy products:

-Why?

+ Tyramine, a potentially potent migraine trigger, is rich in aged cheese.

+ Other common dairy products—yogurt, frozen yogurt, buttermilk, and sour cream—can trigger migraine

+ Milk, cream, butter, and ice cream are usually not a problem

-Substitute with:

+ Young varieties of cheese low in tyramine: cottage cheese, ricotta, cream cheese, and good-quality American cheese.

+ After migraines are under adequate control, you may add fresh goat cheese and mozzarella back to your diet if you desire.

+ BE CAREFUL with cheese on pizza!

6) Nuts

-Why?

+ Pistachios, walnuts, pecans, almonds, coconuts, cashews, Brazil nuts, and nut extracts—contain tyramine, which can trigger migraines.

+ Peanuts (which are actually legumes) and peanut butter should be avoided.

-Substitute with:

+ Sunflower seeds, pumpkin seeds, and any other types of seeds can satisfy that missing nutty flavor!

7) Alcohol and Vinegar

-Why?

+ Too much alcohol in one setting can lead to an accumulated amount of acetaldehyde, a product of the alcohol breakdown. Acetaldehyde triggers the migraine experience known as a hangover. Yes, a hangover is a migraine

+ Congeners, chemicals that develop in the process of fermenting certain alcoholic beverages and give distinctive tastes can cause headaches.

+ Sulfites and phenols in alcohol can trigger migraines

+ Both wine and beer, including non-alcoholic options, contain tyramine and other vasoactive amines, which can trigger migraines.

+ Tap (draft) beers might be higher in tyramine than bottled ones.

+ Different types of vinegar (such as apple cider vinegar) are fermented, including dark ones (such as balsamic, which contain sulfites)

+ Pickled products and condiments (ketchup, mustard, and mayonnaise) that are prepared with non-distilled vinegar should be avoided

-Substitute with:

+ Among all alcoholic beverages, vodka is low in congeners, which can be tolerated in limited quantities.

+ Mocktails with safe ingredients, such as pomegranate juice, cucumber, and mint. Additional recipes can be found at https://thedizzycook.com/

+ Buy distilled vinegar instead.

+ Buy mayonnaise and mustard that is made with distilled vinegar

8) Certain fruits and juices

-Why?

+ Citrus fruits (oranges, lemons, limes, grapefruits, tangerines, clementines, and pineapples) and their juices are healthy foods, but unfortunately, they have been reported to trigger migraines. No need to eliminate vitamin C and citric acid. ***In fact, please make sure to take vitamin C supplements while following the elimination diet***)

+ Bananas can be a potent migraine trigger

+ Dried fruits preserved with sulfites, raisins, raspberries, red plums, passion fruits, figs, dates, papayas, and avocados are rich in tyramine.

+ Not all fruits rich in tyramine will trigger migraines; therefore, you may still be able to tolerate them after slow reintroduction.

-Substitute with:

+ Other types of fruits, such as apples, peaches, pears, berries, cherries, etc (the list provided below)

9) Certain vegetables, especially onions

-Why?

+ Onion is in many prepared foods. Thus it can be difficult to eliminate from the diet

+ Onions, onion powder, sauerkraut, pea pods, certain beans (such as broad, Italian, lima, fava, and navy beans), and lentils can be a migraine dietary trigger

-Substitute with:

+ Pinto and black beans

+ Leeks, scallions, shallots, and spring onions

+ After the elimination stage, you can reintroduce cooked onions (less amount of tyramine) to check your tolerance.

10) Fresh yeast-risen baked goods

-Why?

+ Although young/fresh food is recommended in general, avoid FRESH or HOMEMADE (less than 1 day old) bread, especially sourdough, and yeast-risen bakery items (including bagels, doughnuts, pizza dough, soft pretzels, and coffee cake).

+ Avoid bakery items that contain dried fruits (such as raisins), chocolate, cheese, nuts, bananas, and citrus

+ Avoid croutons and bread crumbs that are seasoned with MSG (read labels)

-Substitute with:

+ packaged bread from grocery stores may be less likely to trigger a migraine

+ You can still have bread that is older than one day

11) Aspartame (Nutrasweet)

-Why?

+ Aspartmate can be a migraine trigger for many individuals. .

-Substitute with:

+ Other artificial sweeteners, such as sorbitol, xylitol, mannitol, and sucralose (Splenda), and sugar itself in moderation are less likely to trigger migraine

+ If you crave diet sodas, you can try caffeine-free diet cola without aspartame, such as Diet Rite (it contains sucralose rather than aspartame)

Each person's response to food triggers is unique. It is impossible to list every personal dietary trigger. For example, some people might have a migraine triggered by tomatoes or tomato-based sauces, mushrooms, peas, or any number of other items. If you suspect any food/drink items, please avoid them, and decide whether or not you want to reintroduce them.

Here's a list of food that you can eat. It is LONGER than the list of prohibited foods. Trust in yourself and trust in the process. There are plenty of recipes that you can make with the listed items.

POWERFUL TIPS:

-Many people with migraines have succeeded with the elimination diet. We are grateful for the founder and author of The Dizzy Cook, Alicia Wolf, who struggled with vestibular migraines over

the years. She developed recipes based on migraine-friendly ingredients/substitutions recommended by Dr. David Buchlolz, M.D. (the author of Heal Your Headache. The Dizzy Cookbook website offers many ideas. Over time, you may be able to create recipes on your own. *(Disclaimer: We are not affiliated with either of the authors listed above or their websites. It is simply a popular and trusted cooking website that strictly follows the Migraine Elimination diet)*

https://thedizzycook.com/category/recipes/

Page 3: Sample Weekly Recipe Guide

- In reality, many people, such as college students, may not have the time and financial resources to cook a different recipe for every meal. Use this weekly recipe grid as a resource when you meal prep for the week. For example, you may choose 2 recipes for breakfast, 2 for lunch, 2 for dinner, and 1 for snacks. You can also choose recipes that share a few or some similar ingredients to save money.

	Mon	Tues	Wed	Thurs	Fri	Sat	Sun
Breakfast	Mango Lassi (without yogurt)	Faux-Yo Acai Bowl without bananas	Burrata Toasts	Vanilla Chia Pudding Parfait	Green Power Smoothie	Buckwheat Pancakes	Pumpkin Seed Smoothie
Snacks	Homemade Cereal Bars	Energy Balls	Trader Joe’s (potatoes snack with only olive oil and salt)	White Chocolate Peppermint Cookies	Cinnamon Oatmeal Muffins	Energy Balls	Cranberry Jalapeno Dip

Lunch	Watermelon Goat Cheese Salad	Turkey Pesto Sandwich	Tuna Pesto Pasta	Pomegranat-e Chicken salad	Tuna Salad	Lemongrass Ginger Soup	Beef Protein Bowl
Dinner	Crockpot Tuscan Chicken	Salmon with Mango Salsa	Steak Taco	Apricot Chicken	Gluten-Free Pad Thai	Healthy Sloppy Joes	Honey Garlic Chicken
Drinks	Winter Wassail	Pomegranat-e Mocktails	Rooibos Latte	Blueberry Mocktail	Warm Cinnamon Milk	Strawberry Mocktail Spritzer	Sparkling Pear Mocktail

Link to more recipes:

https://thedizzycook.com/

Notes:

-The list of migraine-triggering items is long, but not longer than the list of allowed food. Rest assured that you should not lose any macronutrients and important vitamins and minerals while following this diet, especially when the entire elimination diet lasts only two months in total (1 month of the elimination stage + 1 month of the reintroduction stage).

-This elimination diet was created by David Buchholz, M.D. It has been reviewed by several UC Davis physicians. Remember, if you have another significant medical issue besides migraines, you should discuss this approach first with your physician.

-Now that you've read the "Nuts and Bolts" section, try the game again. Did any of the potential substitutions surprise you?

For optimal viewing, you should expand to Full Screen Mode by clicking the icon in the top right of the Nearpod window. You should not need to make an account for any other sites. If you are asked to enter your name for an activity, please use your study ID.

Page 4: Elimination Diet FAQs

- **<u>What to do while on vacation:</u>**
  - Remember the migraine threshold concept: during vacation, a common migraine trigger–stress level–is typically lower. Therefore, your migraine thresholds may be high enough to tolerate not-so-migraine-friendly ingredients, and thereby certain foods may not trigger a migraine.
  - Limiting processed food (such as bacon, etc.) is still recommended; try your best.
  - Limit the *quantity* of prohibited foods if possible
  - Of course, avoid ingredients that you know personally trigger your migraine symptoms
- **<u>What to do when at an airport:</u>**
  - There are many factors that you cannot avoid, such as barometric pressure changes, weather, airport food, long flight, etc. Therefore, do your best, and don't be too hard on yourself. .
  - Bring migraine-safe snacks
  - Choose food that has more migraine-friendly ingredients and remove the migraine-triggering ingredients from your food as best as you can.
  - Continue to follow the previously mentioned recommendations for limiting processed foods, eating small amounts of the prohibited foods, and avoiding personal food triggers.
  -
- **<u>What to do with food from outside soucres (events, restaurants, other people's houses):</u>**
  - Communicate with others that you are on a diet
  - Remember, do not expect to be perfect in eliminating every single possible food trigger in every situation.
  - Provide your hosts and others ahead of time with a list of ingredients that cause your flare-ups

- Continue to follow the previously mentioned recommendations for limiting processed foods, eating small amounts of the prohibited foods, avoiding personal food triggers / removing migraine-triggering ingredients as best as you can.

- **What to do when loved one(s) or others share the same food, and/or you have to eat what's offered to you:**

  - Communicate thoroughly with your loved ones or others about your migraines and the benefits of doing the elimination diet.

  - Ask them if they want to do a less intense version of the diet (by limiting MSG, processed food, fast food, and restaurant food). This can be a fun way of keeping each other accountable.

  - Remind them that the elimination diet is only temporary; the purpose is to find what ingredients might trigger migraines.

- **What to do about the cost limiting factor of fresh food:**

  - The elimination diet requires fresh food and seasonings that can be expensive and unsustainable for college /graduate students. However, the diet also helps save money by avoiding eating out or buying coffee /processed food. Cooking from home can be a great way to save money.

  - Many colleges, especially UC Davis, have programs that support students. Please check out the below links:

    - https://www.ucdavis.edu/news/food-access-and-security#:~:text=Students%20can%20be%20eligible%20for,the%20Memorial%20Union%20Information%20Desk.

    - https://studentparents.ucdavis.edu/financial

  - The city of Davis has food bank options: https://yolofoodbank.org/find-food/

  - Make sure to check if you are qualified for Calfresh EBT (Electronic Benefits Transfer) benefits

  - If you are not qualified for any programs and don't have the financial resources, try your best not to eat out and use the money to cook at home instead. Use guidelines from the Dizzy cookbook or our example to create meals that fit your budget.

- **How to stick to this elimination diet when limited on time:**
    - It's important to learn how to manage time well. That means study time should be protected from social media and other forms of distraction.
    - A couple of tips on time management:
        - Take frequent breaks to avoid energy dips
        - Try the Pomodoro technique https://todoist.com/productivity-methods/pomodoro-technique
        - Use the longer breaks to prepare ingredients for cooking
        - Do grocery shopping and meal prep on weekends
        - Clean as you go to avoid spending excess time cleaning up later
        - Meal prep smoothies and freeze them.
    - Improving time management skills may help with finding time to cook. Know your priorities and do your best to achieve them.
    - If you have to work multiple jobs to pay the bills as a student, even with excellent time management skills, you may have very little time to rest or cook. Try your best to then limit prohibited food (ex: fast food, processed food, etc.), obtain at least 7 hours of sleep each night, and 10 minutes of aerobic exercise daily to improve the migraine threshold and lower the total trigger load.
- **This diet seems so high maintenance. How can one maintain the diet over the years?**
    - You don't have to do the elimination diet for the rest of your life. The elimination diet's purpose is to discover your food triggers, if there are any. You can reintroduce the foods that are not triggering and make conscious choices about when or if you want to eat any triggering foods.
    - A typical migraine treatment plan involves multiple aspects to help decrease migraine over time. We encourage people to have active participation in their migraine treatment plan and learn how to control avoidable triggers as best as possible. Part of the plan can include the elimination diet, so try your best!

-Look at the ingredients in this chicken broth. Based on what you have learned so far, do you think this broth is safe from all possible migraine triggers? If not, what are the potential triggers that it contains?

Read the next module to find out the answer!

Page 5: Tips for Reading Food Labels

Disclaimer: This is not a guide on how to read labels and determine appropriate nutritional portions such as macronutrients and micronutrients. Instead, we aim to show you some useful rules for reading ingredients.

Rules of thumb:

1) Ingredients are listed in decreasing order. For example, if an onion is listed as the second-to-last ingredient, it might be safe. However, if it's listed as the second or third ingredient, you might not want to consume it during the elimination stage.
2) Organic-labeled food may not be safe if it contains possible trigger ingredients.
3) Processed meats with safe ingredients and without nitrites/nitrates may not be safe because they are processed and modified, especially during the elimination stage.

Food Examples:

(Separate Document)

Other useful resources:

https://www.henryford.com/blog/2016/12/easy-tips-to-understand-the-ingredient-list-on-food-labels

https://www.healthline.com/nutrition/how-to-read-food-labels

https://www.heart.org/en/healthy-living/healthy-eating/eat-smart/nutrition-basics/understanding-ingredients-on-food-labels

24 Hour Diet Recall Revisited

-Using what you learned about potential migraine triggers and substitutions, update the 24 Hour Diet Recall table that you made in Module 1. Strike through the items you wrote that are potential migraine triggers. Underneath, add new items that you could use to substitute for potential triggers. Highlight new items in yellow. For example:

Time	Meal	Ingredients
breakfast	omelet	Eggs
		Butter
		Milk
		~~Cheddar cheese~~
		American cheese
snack	~~orange~~	-
	strawberries	

This is for your own educational purposes. You do not need to submit your document to Canvas.

Page 6: Bonus Tips and Resources

++ Dietary tips:

1) Be prepared that caffeine withdrawal can trigger migraines, and last a few weeks. It is recommended to wean caffeine slowly and then stop it, to help avoid this scenario as much as possible. The long-term benefits of caffeine avoidance can be worth it.

2) Monosodium glutamate (MSG) is a common ingredient that is often disguised as different names (see earlier section). Read labels.

3) Try your best to eat fresh food (except fresh baked goods)

4) By substituting MSG with a diverse range of spices and seasonings, you may be surprised by the amazing flavor achieved.

5) You can have ALL sources of proteins, including fresh meat (not processed), poultry, and seafood.

6) If you do get a migraine triggered by eating one of the foods that are supposed to be eliminated, have compassion for yourself! You can start again. Don't give up. The first step to building a habit is recognizing mistakes. You are still in CONTROL!

++ TIPS on having a successful elimination diet:

1) No one can follow the elimination diet perfectly. It can be very difficult to avoid all triggers due to other factors (financial constraints, school, job demands, etc.). Nonetheless, there are things that you can do personally to reduce the risk of having a migraine.

2) If your goal is to reduce migraines with little or no medications, discovering your food triggers can be a valuable tool.

3) Positive mindset: view this diet as an opportunity to have a clean and healthy diet for your overall health. Many ingredients on the prohibited list are pro-inflammatory and/or unhealthy. This diet also provides an opportunity to practice self-discipline!

4) While the list of potential triggering foods is long, there are still many foods that you can eat. We encourage you to focus on the foods that you can have. Additionally, you don't have to avoid all of the items listed as potential food triggers for the rest of your life. The purpose of doing the elimination stage is to discover your personal food triggers.

5) GOOD NEWS: It can be tough to follow the diet initially, especially if you're already on a restricted diet (for example: vegetarian, etc.). So when your headaches have been adequately controlled for a period of time, you may be able to tolerate small

quantities of food triggers. You can choose to dial up or dial down the food, depending on your tolerance to each item

6) At the beginning of this elimination diet, you should avoid ALL food triggers so that you can get a good understanding of what foods are triggering for you.

Bonus Tips and Resources:

-This tips section is a little long, you might want to break it in half (have a Part 1 and Part 2)

Bonus tips (including tips designed for students):

1) Communicate with your friends and family that you are on an elimination diet. If you choose to have food prepared by restaurants or at someone else's house, try your best to eliminate potential food triggers (for instance: you can order a taco salad without beans, onions, and tomatoes).

Communicate with your roommates and housemates about having a dedicated space in your fridge to help avoid conflicts.

2) If you live with others, encourage them to try a less intense version of this elimination diet with you. Make it fun! A less intense version can simply exclude caffeine, junk food / fast food, processed food, and food rich in MSG.

3) Consistently remind yourself that you are doing this diet to reduce future migraine attacks. Also, remind yourself that this elimination diet will only be temporary.

4) Meal prep on weekends, or during your long studying breaks.

5) If school and job events offer free food (for instance, pizza), try your best not to eat them. You don't know the ingredients in the food and it may work against the elimination diet.

6) Don't be afraid to ask restaurants, friends, etc, what ingredients are in the food. If they are not sure, try avoiding it.

++ **Getting in the Right State of Mind**

OTHER lifestyle approaches that are highly encouraged to follow to achieve a higher migraine threshold:

1) Exercise: recommendations state you should exercise for about 20 minutes daily. Exercise creates endorphins that can elevate the migraine threshold, and typically last for 24 hours, thus, daily exercise is necessary to maintain that level. It is also important to avoid overexertion (for example: a brisk walk, or low-impact aerobic classes can be beneficial). Side bonus: exercise helps you sleep!

2) Reflect on your values and prioritize habits that nourish your values. For instance, as a medical student who also cares for two younger sisters, my schedule is packed with school and life responsibilities. But to be a good physician, daughter, sister, mother, friend, etc., I must first take the best care of my health. Without good health, I cannot fulfill my life duties

3) Create healthy boundaries: it is ok to say "no." Communicate with others about your needs, and accept that you cannot care for everyone's problems. Here's a useful resource to learn more about this skill: https://psychcentral.com/lib/10-way-to-build-and-preserve-better-boundaries

4) Maintain good sleep hygiene: wake up and go to sleep at the same time every day, including weekends. Sleep hygiene is recommended: for example, blackout curtains to decrease the light in the room; avoid electronic devices at least 1 hour before bedtime; stretch before bedtime; have a good sleeping posture with a pillow underneath your knees if you sleep on your back or in between the knees if you sleep on your side; get sunlight to absorb vitamin D through your eyes early in the morning. Here is a book for how to improve sleep hygienehttps://www.amazon.com/Sleep-Smarter-Essential-Strategies-Success/dp/1781808368/ref=asc_df_1781808368/?tag=hyprod-20&linkCode=df0&hvadid=312403172530&hvpos=&hvnetw=g&hvrand=12024067483274590957&hvpone=&hvptwo=&hvqmt=&hvdev=c&hvdvcmdl=&hvlocint=&hvlocphy=9032532&hvtargid=pla-465874675151&psc=1&tag=&ref=&adgrpid=60300244057&hvpone=&hvptwo=&hvadid=312403172530&hvpos=&hvnetw=g&hvrand=12024067483274590957&hvqmt=&hvdev=c&hvdvcmdl=&hvlocint=&hvlocphy=9032532&hvtargid=pla-465874675151

5) Ask your doctor about supplements that have been well studied in decreasing the frequency of migraines:

a) Vitamin B2

b) Magnesium

c) CoQ10

6) As a student, school, internships, and other extracurricular actvities can take up the majority of our time. Many of us might end up sacrificing sleep and other healthy habits to put in a few more hours of studying. Therefore, time management is a crucial skill. Here is a book on how to study effectively:https://www.amazon.com/Make-It-Stick-Peter-C-Brown-audiobook/dp/B00M1Z2THY/ref=sr_1_1?crid=1J3FUM2YKSLW&keywords=make+it+stick&qid=1673333911&s=books&sprefix=make+it+stic%2Cstripbooks%2C295&sr=1-1

7) Mental well-being (victimization and dependence): people with chronic conditions, including migraines, can have low self-efficacy. This concept explains that people with chronic conditions may believe they lack capacity to execute behaviors necessary to produce specific performance goals. People with migraines, over time, might succumb to being victimized by constant migraines instead of trying various techniques to prevent migraines. There can be a higher tendency to think you have no control over the condition. Medical research shows that people with certain chronic conditions, such as migraine, can be more prone to develop mental health comorbidities such as anxiety and depression. Sometimes it is easy to be anxious about another migraine attack impacting our careers, dreams, and life (this can lead to a concept known as catastrophic thinking).

8) Seek therapy: It's crucial to learn coping tools and strategies for how to rewire distorted thoughts, especially during a flare-up. Stress / mental health management with certain techniques (for example, cognitive behavior therapy, mindfulness, etc.) can retrain your brain, thoughts, and body to react to certain situations with less of the "catastrophic thinking effect," thereby improving quality of life. It takes time to have a brain wired to react a certain way during a chronic condition, thus, it takes time to achieve the retraining. The more you practice the elimination/reintroduction diet skills over time, especially on the "good" days (low total trigger load), the easier it will be for you to use these skills on the "bad" migraine days (high total trigger load). This same practice recommendation applies to other coping skills techniques such as yoga/meditation, deep breathing, etc. Here is the website for UC Davis student counseling options:

https://shcs.ucdavis.edu/services/counseling/community-advising-network

9) These books may offer additional insight:

- When things fall apart https://www.amazon.com/When-Things-Fall-Apart-Difficult/dp/B0BR6125KY/ref=sr_1_1?crid=30DUW1NKA7F28&keywords=when+things+fall+apart&qid=1673334674&s=audible&sprefix=when+things+fall+apar%2Caudible%2C195&sr=1-1

- The body keeps the score https://www.amazon.com/Body-Keeps-Score-Healing-Trauma/dp/B08TX585RN/ref=sr_1_1?crid=2JKAKWMSMM2KU&keywords=Body+keeps+score&qid=1673334712&s=audible&sprefix=body+keeps+scor%2Caudible%2C276&sr=1-1

- The Power of Now

Table S1. Knowledge and Self-Efficacy at Time 2 (n = 33)

	Completely disagree n (%)	Somewhat disagree n (%)	Neutral n (%)	Somewhat agree n (%)	Completely agree n (%)
**Knowledge**					
1. I feel that I have been well-informed about ways in which I can be most successful with the elimination diet.	0 (0.0)	0 (0.0)	2 (6.1)	15 (45.5)	16 (48.5)
2. In general, the education from this study has helped me improve my diet.	0 (0.0)	0 (0.0)	6 (18.8)	15 (46.9)	11 (34.4)
3. In general, my nutrition knowledge has improved since being on the elimination diet.	1 (3.2)	1 (3.2)	5 (16.1)	14 (45.2)	10 (32.3)
**Self-Efficacy**					
1. Following the elimination diet has improved my eating habits in general.	1 (3.3)	1 (3.3)	6 (20.0)	12 (40.0)	10 (33.3)
2. I find the benefits of the elimination diet for improving my migraine symptoms outweigh the inconveniences that come with having a restricted diet.	0 (0.0)	7 (23.3)	4 (13.3)	11 (36.7)	8 (26.7)
3. I would recommend the elimination diet to other people with migraines.	1 (3.2)	0 (0.0)	3 (9.7)	16 (51.6)	11 (35.5)
4. The elimination diet does a good job in keeping my migraine symptoms under control.	3 (10.7)	0 (0.0)	11(39.3)	7 (25.0)	7 (25.0)

Table S2. Change in MIDAS Scores by Medication Change

MIDAS Score	Baseline Mean (SD)	Time 2 Mean (SD)	Difference Mean (SD)	P Value
**Full Sample**				
All participants (n = 33)	42.7 (6.7)	28.5 (3.8)	-14.2 (8.4)	.100
No new medications (n = 25)	45.1 (8.2)	29.5 (4.7)	-15.6 (10.8)	.161
Yes new medications (n = 8)	35.0 (8.4)	25.6 (5.6)	-9.4 (8.7)	.317
Difference of differences (Yes vs No)			-6.2 (19.8)	.756
**High Adherence**				
All (n = 19)	43.3 (8.3)	25.1 (4.8)	-18.3 (11.0)	.114
No new medications (n = 15)	42.4 (10.0)	25.5 (5.7)	-16.9 (13.9)	.242
Yes new medications (n = 4)	46.8 (14.4)	23.5 (9.0)	-23.3 (8.2)	.065
Difference of differences (Yes vs No)			6.3 (26.7)	.822
**Low Adherence**				
All (n = 14)	41.8 (10.9)	33.4 (6.1)	-8.4 (13.3)	.536
No new medications (n = 10)	49.2 (14.7)	35.6 (8.1)	-13.6 (18.0)	.469
Yes new medications (n = 4)	23.3 (5.6)	27.8 (8.2)	4.5 (12.6)	.745
Difference of differences (Yes vs No)			-18.1 (30.1)	.559

Note. High Adherence = Adherence score 4 (somewhat agree) or 5 (completely agree)

Low Adherence = Adherence score 1 (completely disagree), 2 (somewhat disagree, or 3 (neutral)

Sample is divided into those who did not start new medications while in the study (No) vs. those who started a new medication while in the study (Yes).

Table S3. Barriers to Implementing the Elimination Diet at Time 2

	Completely disagree (%)	Somewhat disagree (%)	Neutral (%)	Somewhat agree (%)	Completely agree (%)
**Social-Emotional** (*n=30)					
1. Social situations, like going out with friends or family, make it harder for me to follow the elimination diet.	7.4	3.7	18.5	37.0	33.3
2. Following the elimination diet makes me anxious.	14.8	40.7	18.5	18.5	7.4
3. When my symptoms are in control, I sometimes stop following the diet more than usual.	3.7	22.2	14.8	55.6	3.7
4. Following the elimination diet makes me feel down or depressed.	29.6	40.7	7.4	18.5	3.7
5. When I am stressed or feeling ill, I stop following the elimination diet more than usual.	0.0	22.2	3.7	55.6	18.5
6. I find the elimination diet to be much more difficult than I expected it to be.	3.0	40.7	18.5	22.2	11.1
7. Traveling for pleasure/work has introduced a challenge in following the elimination diet.	7.4	11.1	29.6	25.9	25.9
**Cost and Convenience** (*n=29)					
With regard to following the elimination diet, it can be difficult to...					
1. Find something to eat.	22.2	14.8	22.2	13.6	3.0
2. Eat out for fear of contamination.	29.6	25.9	11.1	22.2	11.1
3. Spend a lot of time planning meals.	3.7	22.2	14.8	29.6	29.6
4. Read food labels and shop and special stores.	25.9	29.6	22.2	14.8	18.5
5. Stick to my grocery budget.	29.6	14.8	22.2	14.8	18.5
6. Find foods I can eat because of my migraines.	11.1	29.6	22.2	25.9	11.1
7. Follow because it's expensive.	22.2	40.7	11.1	14.8	11.1
8. Follow because it's restrictive.	7.4	18.5	18.5	40.7	14.8
9. Throw away and/or donate my food and ingredients that are potential triggers.	23.1	23.1	11.5	42.3	0.0
10. Plan out my meals, read labels, and cook meals with specific restrictions due to school and life responsibilities.	7.4	7.4	22.2	37.0	25.9
11. Implement the elimination diet because of the fixed student meal plan.	40.7	11.1	37.0	7.4	3.7

* Participants who did not attempt the elimination diet were instructed to skip these questions. Of the 33 participants who responded to the Time 2 questionnaires, only 30 participants answered the social-emotional questions and only 29 answered the cost and convenience questions.
